# Transitional Flow in a Cylindrical Flow Chamber for Studies at the Cellular Level

**DOI:** 10.1007/s13239-012-0107-5

**Published:** 2012-09-11

**Authors:** Susan M. McCormick, Justin T. Seil, David S. Smith, Francis Tan, Francis Loth

**Affiliations:** 1 Section of Vascular Surgery and Endovascular Therapy, Department of Surgery, University of Chicago, MC 5028, 5841 S. Maryland Ave., Chicago, IL 60637 USA; 2 Department of Bioengineering, University of Illinois at Chicago, Chicago, IL USA; 3 Engineering Health and Sciences Division, College of Du Page, Chicago, IL USA; 4 Departments of Mechanical and Biomedical Engineering, University of Akron, Akron, OH USA

**Keywords:** Endothelial cells, Arterio-venous grafts, Transitional flow, Morphology, WSS, Reynolds number

## Abstract

Fluid shear stress is an important regulator of vascular and endothelial cell (EC) functions. Its effect is dependent not only on magnitude but also on flow type. Although laminar flow predominates in the vasculature, transitional flow can occur and is thought to play a role in vascular diseases. While a great deal is known about the mechanisms and signaling cascades through which laminar shear stress regulates cells, little is known on how transitional shear stress regulates cells. To better understand the response of endothelial cells to transitional shear stress, a novel cylindrical flow chamber was designed to expose endothelial cells to a transitional flow environment similar to that found *in vivo*. The velocity profiles within the transitional flow chamber at Reynolds numbers 2200 and 3000 were measured using laser Doppler anemometry (LDA). At both Reynolds numbers, the velocity profiles are blunt (non-parabolic) with fluctuations larger than 5% of the velocity at the center of the pipe indicating the flows are transitional. Based on near wall velocity measurements and well established data for flow at these Reynolds numbers, the wall shear stress was estimated to be 3–4 and 5–6 dynes/cm^2^ for Reynolds number 2200 and 3000, respectively. In contrast to laminar shear stress, no cell alignment was observed under transitional shear stress at both Reynolds numbers. However, transitional shear stress at the higher Reynolds number caused cell elongation similar to that of laminar shear stress at 3 dynes/cm^2^. The fluctuating component of the wall shear stress may be responsible for these differences. The transitional flow chamber will facilitate cellular studies to identify the mechanisms through which transitional shear stress alters EC biology, which will assist in the development of vascular therapeutic treatments.

## Introduction

One of the major regulators of vascular and endothelial cell (EC) function is fluid shear stress. Among the effected functions are vascular tone, remodeling, cell migration, platelet adhesion, permeability, and the immune/inflammatory system. However, its effect on cells depends not only upon its magnitude but also upon the type of flow creating the shear stress. Based on complexity, flows can be divided into three regimes: (1) laminar where fluid flow is smooth and repeatable, (2) turbulent where flow is continually chaotic with eddies and vortices of many scales, and (3) transitional which is somewhere between turbulent and laminar flow. In transitional flows there are fluctuations of velocity and pressure which are not repeatable.^35^ The dimensionless Reynolds number (Re = 4ρ*Q*/π*μD* where *Q* is the flow rate, ρ is the density, μ is the dynamic viscosity and *D* is the diameter) is used to characterize these flow regimes (for straight tubes: laminar Re < 2200, turbulent Re > 10,000, transitional 2200 < Re < 10,000). The differences in the velocity patterns of transitional and laminar flow account for the unique effects of the resulting shear stresses on cells.

Transitional flow has a random dynamic multidirectional flow component that is superimposed over orderly flow, while laminar flow is well ordered with fluid particles traveling in non-intermixing layers. Because transitional flow is disorganized, the shear stress the cells are exposed to randomly fluctuates in magnitude and direction.^29^ In contrast, under steady laminar flow conditions shear stress direction is non-varying. *In vivo*, pulsatility can cause the direction of laminar shear stresses to oscillate. However the frequency of these oscillations is close to the heart rate (1 Hz), while transitional fluctuations are at a higher frequency (40–300 Hz).^22^,^29^,^32^

ECs discriminate between different shear stresses with their response depending upon the fluid flow profile. For example turbulent flow shear stresses increase EC proliferation rates,^7^ whereas laminar shear stresses inhibit the *G*_0_/*G*_1_ to *S* phase transition.^1^ One hundred genes were identified by Garcia-Cardena *et al*.^15^ to be differentially regulated by laminar and turbulent shear stress. The random component of transitional shear stress results in the cells being exposed to a greater number of spatial and temporal shear stress gradients in comparison to laminar shear stress. Several studies indicate that ECs detect these gradients and that they play an important role in the shear stress cellular response.^2^,^9^,^34^

In the vasculature, flow profile and shear stress magnitude vary with location. In the majority of the vessels the flow is laminar. Disturbed flow characterized by secondary flows, vortices and intermittent changes in flow direction can be present in curved and bifurcated vessels like the coronary and carotid arteries.^11^,^16^,^17^,^33^ The physiological significance of disturbed flow and the corresponding shear stresses that are sensed by ECs is evident from their correlation with vascular diseases. The most notable is the co-localization of early atherosclerotic lesions with disturbed flow in curved and branching arteries, and the lack of these lesions in regions where there is high laminar shear stress.^20^,^21^,^36^ Although turbulent flow can occur during times of increased blood flow it is rarely present *in vivo*.^3^ Transitional flow often develops downstream of stenotic regions.^23^,^32^,^43^,^44^,^48^ Currently, the EC response to a given shear stress magnitude in transitional flow compared to that in laminar flow is not well understood.

Several systems have been developed and used to study the effects of different types of shear stress on cells *in vitro*. The effects of laminar shear stress on ECs have been studied primarily using either a parallel plate flow chamber or a cone and plate viscometer to impose the mechanical stress. The parallel plate flow chamber consists of a slide of cells sealed to a base.^14^,^26^ There is a narrow channel between them through which media flows to expose the cells to shear stress. In the cone and plate system, a cone is rotated about an axis perpendicular to the surface of a Petri dish causing concentric fluid movement which exposes cells to shear stress.^4^,^10^ This apparatus is also used to expose cells to turbulent shear stress. A modified Reynolds number was defined by Sdougos *et al*.

$$ \overline{R} \equiv \frac{{r^{2} \omega \alpha^{2} }}{12\nu } $$

where *r* is the radial distance from the apex of the cone, ω is the angular velocity of the cone, α the cone angle and ν the kinematic viscosity of the fluid to characterize the flow in a cone and plate system.^42^ Their measurements demonstrated that laminar flow occurs when *R* < 1 and turbulent flow exists when *R* > 4. To study the effect of disturbed flow on cells, many investigators use a parallel plate flow chamber that has been modified to include a rectangular step.^6^ The step is perpendicular to the direction of flow and spans the width of the chamber. Regions of flow separation, recirculation (a steady two-dimensional vortex), attachment and recovery have been identified in these systems using finite element analysis and flow visualization. Experiments utilizing these systems have Reynolds numbers less than 500 which is in the laminar region. Thus, the flow present in the channel is disturbed laminar flow.

We have developed and characterized a cylindrical flow chamber to study the effect of transitional flow. In particular, it was designed so the Reynolds number could be matched to the Reynolds numbers for flows in arterio-venous grafts (AVGs). AVGs are created in individuals with end stage renal disease for vascular access during dialysis. Unfortunately, their lifespan can be relatively short. At 3 years, 80% of AVGs have required interventions (thrombolysis, elective angioplasty or surgery) and 50% of AVGs have been abandoned or removed. Although thrombotic episodes precipitate 80% of vascular access failures, the underlying primary cause of failure is stenosis^41^ with more than 80% of these lesions occurring in the vein at or immediately downstream of the venous anastomosis (venous–graft junction).^19^ Patients with AVGs have Reynolds numbers between 1000 and 3000.^38^ Based on experimental and computational fluid dynamic simulations, flow in AVGs is transitional with complex velocity profiles and fluctuating velocities and pressures.^22^,^29^ Fillinger *et al*.^12^ have shown a correlation between graft Reynolds number and intimal hyperplasia in a canine model.

Several investigators have shown that the effects of shear stress depend not only on its magnitude but also on the type of flow that created it.^5^ Thus, it is expected that the effects of transitional shear stress will be different from those of laminar, disturbed and turbulent. In agreement with this there are differences in the diseases that occur at locations of disturbed and transitional flow. At locations of disturbed flow there is an increase in the rate of atherosclerosis, whereas as in AVGs with transitional flow there is intimal hyperplasia (IH). The pathogenesis of AVG IH differs from atherosclerosis and restenosis. In AVG IH there are very few macrophages, monocytes and lymphocytes and no foam cells or lipid deposits that are present in atherosclerotic lesions.^45^ In addition, smooth muscle cell proliferation rates are significantly greater in stenotic AVG veins than in atherosclerotic vessels.^39^ Moreover, the development of arterial restenosis after angioplasty differs from that of AVG stenosis. Proliferation of smooth muscle cells at restenotic sites occurs primarily within the first few days, whereas in AVG lesions there is a more continuous proliferation.^45^ Additionally, the patency rate of AVGs that have had angioplastic interventions is lower than that of atherosclerotic vessels after similar treatments. This suggests that different factors are contributing to IH in these two regions.^40^

This paper describes a cylindrical flow chamber specifically designed to study the effect of transitional flow at the cellular level. The flow environment within the chamber was carefully characterized. The system was then used to study the effect of transitional shear stress on EC morphology. Although this system was designed to study the effects of AVG transitional flows on cells, it can also be utilized to study transitional flows downstream of a stenosis.

## Materials and Methods

### Design of Transitional Flow System

**Figure 1:**
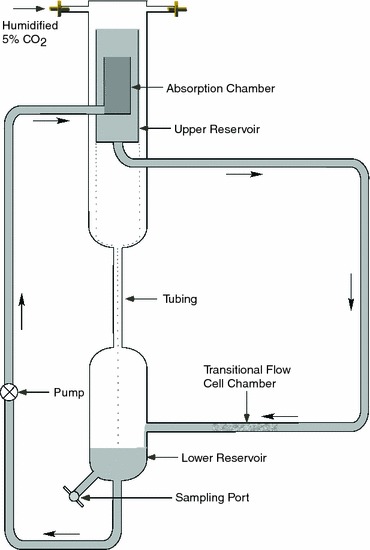
Transitional flow system. Cells are precultured on a polycarbonate film that is inserted into the cell chamber for exposure to transitional shear stress. Media flows from the upper reservoir to the cell chamber due to hydrostatic pressure. After flowing through the cell chamber the media flows into the lower reservoir from which it is pumped to the absorption chamber with a peristaltic pump. The absorption chamber, which overflows into the upper reservoir, dampens the momentum of the media so that flow to the lower reservoir is driven only by hydrostatic pressure

The cell chamber consists of a glass tube (0.6 cm inner diameter, 19 cm in length). Cells are cultured on a polycarbonate film (7.5 × 1.9 × 0.013 cm), which is curled into a cylinder and inserted into the chamber and positioned near the end of the glass tube. The elastic recoil of the polycarbonate film holds it firmly against the wall of the chamber. There is no movement of the film after it is manually placed in the chamber. The chamber is connected to a recirculating constant hydrostatic pressure flow system (Fig. 1). The inlet tubing is kept in line with the flow chamber to reduce skewing of the velocity profiles due to curvature. In the recirculating flow system, media is pumped from the lower reservoir to the absorption chamber submerged in the upper reservoir. This chamber absorbs the momentum of the fluid that results from it being pumped from the lower reservoir. This ensures that the fluid flow to the cell chamber is driven strictly by the hydrostatic pressure drop between the top of the upper reservoir and the return port of the lower reservoir. To maintain a constant pressure head, pump speed is adjusted so that the upper reservoir is constantly overflowing into the lower reservoir. The distance between the upper and lower reservoirs can be adjusted to change the flow rate.

### Characterization of Transitional Flow Chamber with LDA

Detailed velocity measurements were conducted at Reynolds numbers 2200 and 3000, with flow rates of 400 and 550 cm^3^/min, respectively, in a cell chamber with a diameter of 0.6 cm, using a fluid with a density of 0.995 g/cm^3^ and a dynamic viscosity of 0.65 cP at 37 °C. Measurements were made 9.5 cm from the end, the midpoint of the glass tube providing an entrance length of 9.5 cm (16 diameters). For turbulent flow, the entrance lengths for fully developed flow at Reynolds numbers 2200 and 3000 are 15.9 and 16.7 diameters (*l*_e_/*D* = 4.4Re^1/6^). All velocity measurements were taken with a one-component laser Doppler anemometry system (MiniLDV-100w-660-FS25-3mB, Viosense Corporation, Pasadena, CA) in backscatter mode. The system used a diode laser with a wavelength of 660 nm. The probe volume had a diameter of approximately 40 *μ*m with an axial length of 200 *μ*m. The particles used to scatter the light were 12 *μ*m diameter silver coated glass spheres (SH400S33, Potters Industries Inc., Carlstadt, NJ). The axial velocity was measured at 46 locations across the diameter of the cross section spaced 0.0125 cm apart. At radial locations far from the walls, a velocity measurement was the average value of approximately 900 data points (bursts). Near wall measurements had less data points due to artifacts caused by the wall and the data rate was lower on the far wall compared to the near wall. Data rates for velocity measurements at the near and far walls were between 92–476 and 19–31, respectively. Wall shear stress (WSS) was estimated using the measured wall velocity gradient and classical methods for predicting friction factors for flow in a straight pipe at these transitional flow Reynolds numbers. It is not possible to precisely calculate the time averaged WSS along the region where the endothelial cells are placed. However, WSS can be estimated based on the velocity measurements. The time averaged WSS magnitude will be larger than that of laminar flow and smaller than that of fully turbulent flow. WSS for laminar flow in a straight pipe for the two Reynolds numbers examined are 2.0 and 2.8 dynes/cm^2^, respectively based on Poiseuille flow profile:

$$ \tau = \frac{4\mu Q}{{\pi R^{3} }} $$

where *μ* is kinematic viscosity, *Q* is volume flow rate, and *R* is pipe radius. Surface roughness for the polycarbonate sheet with ECs is approximately 0.0013 (ε = 4 μm, measured from cell junction to maximum cell height^28^). Thus, the turbulent friction factor from the Moody diagram for this surface’s roughness is 0.053 and 0.047 for Reynolds numbers 2200 and 3000, respectively. The relationship between friction factor and time averaged WSS is

$$ \tau = \frac{8f}{{\rho U^{2} }} $$

where ρ is fluid density, *f* is the friction factor and *U* is the mean velocity in the straight pipe. Thus, the time averaged turbulent WSS for flow in a straight pipe at Reynolds number 2200 and 3000 is 3.7 and 6.1 dynes/cm^2^, respectively. Thus, WSS for this transitional flow is expected to be in between the values for laminar and turbulent flow (2.0 < WSS_Re=2200_ < 3.7, 2.8 < WSS_Re=3000_ < 6.1, units are dynes/cm^2^).

### Endothelial Cell Culture and Exposure to Shear Stress

Human umbilical vein ECs (Sciencell, San Diego, CA) were cultured in M199 supplemented with 10% fetal bovine serum (FBS), penicillin (20 units/mL), streptomycin (20 *μ*g/mL), heparin (100 *μ*g/mL) and endothelial cell growth supplement (50 *μ*g/mL). They were incubated at 37 °C in the presence of humidified 5% CO_2_ and 95% air. For exposure to shear stress ECs, passage 3–5 were seeded on polycarbonate slides (laminar shear stress) and film (transitional flow) coated with fibronectin (2 *μ*g/cm^2^) at a density of 5.0 × 10^4^ cells/cm^2^. Note that there was an abrupt change in glass tube lumen with the film in place as it has a thickness of 130 *μ*m. Cells were exposed to laminar shear stresses of 3 and 20 dyne/cm^2^ using a parallel plate flow chamber with a flow surface area of 5.5 × 2.5 cm, inserted into a constant pressure drop flow system.^14^ The characterized cylindrical flow chamber was used for exposure to shear stresses under transitional flow with Reynolds numbers of 2200 and 3000. No LDA measurements were made while the ECs were exposed to shear stress.

### Morphological Analysis

Four random regions were imaged per slide with a CCD camera (SPOT RT Slider, Model 2.3.1, Diagnostic Instruments, Sterling Heights, MI) mounted on a microscope (Nikon Eclipse E600, Nikon Precision Inc., Belmont, CA). Images, 1600 × 1200 pixels, were taken at 100× magnification. In each image, the contours of at least 25 cells were manually outlined for analysis using NIH ImageJ software (http://rsb.info.nih.gov/ij/). Three morphological parameters: cell area, shape index and cell alignment were quantified. Shape index is defined as 4π*A*/*P* ^2^ where *A* is cell area and *P* is the perimeter of the cell. This parameter is used to quantify the shape of a cell in terms of elongation and roundness. The shape index is near zero for elongated cells and close to one for round cells. Cell alignment is defined as the angle between the major axis of the ellipse fitted to the cellular trace and a given direction. For cells exposed to shear stress it was defined as the angle between the major cell axis and the axis perpendicular to fluid flow. Thus, an angle of 0° or 180° would indicate that the cell is aligned perpendicular to the direction of flow and an angle of 90° would mean the cell is aligned parallel to the direction of flow. For cells cultured in the absence of shear stress, the angle was measured between the major axis of the cell and the long axis of the slide. Morphological measurements were performed at 24 and 48 h. Three to four experiments were completed for each time point. A minimum of 100 cells were analyzed per culture condition, per experiment.

### Statistical Methods

Data are expressed as mean ± standard error of the mean (SEM). Each experiment was repeated at least three times. Data was analyzed using one-way ANOVA with the Tukey *post hoc* test. For all tests, statistical significance was defined as *p* < 0.05.

## Results

### Characterization of the Biomechanical Environment in the Transitional Flow System

**Figure 2:**
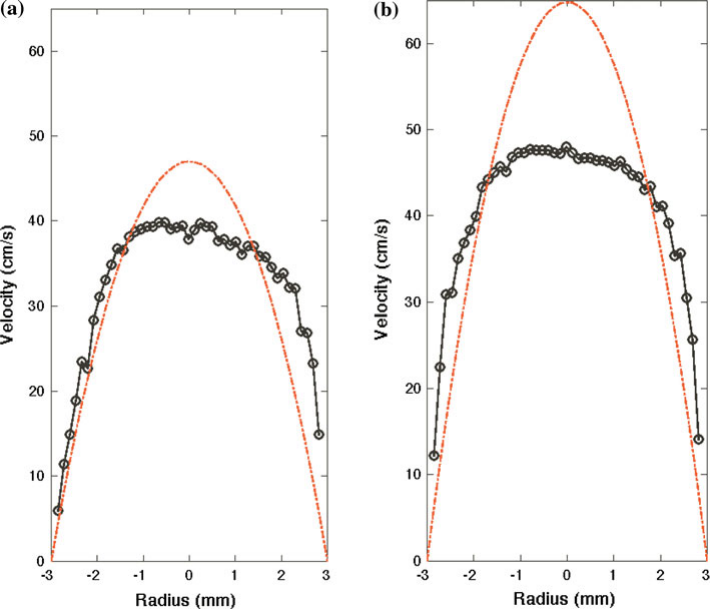
Measurements of the time averaged axial velocity distribution for Reynolds numbers (a) 2200 and (b) 3000 taken with LDA. The theoretical Poiseuille parabolic velocity profile is also shown for each flow rate (dashed lines) to demonstrate the difference between velocity profiles at transitional and laminar flows

**Figure 3:**
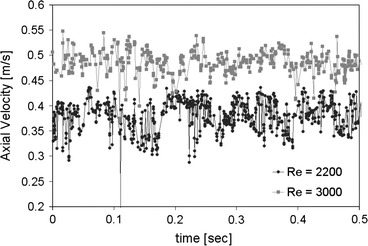
Velocity time histories at the center of the chamber cross-section taken with LDA demonstrating velocity fluctuations due to transitional flow

Measurements of the time averaged velocity profile within the transitional flow chamber at Reynolds numbers 2200 and 3000 were taken using LDA (Figs. 2a, 2b). These results show the velocity profiles to be blunt (non-parabolic) at both Reynolds numbers. The Reynolds number 3000 case is more blunt than that of Reynolds number 2200. The maximum velocity for Reynolds number 2200 and 3000 are 40 and 48 cm/s, respectively. The ratios of maximum velocity to mean velocity for Reynolds number 2200 and 3000 are 1.7 and 1.5, respectively. The ratio of maximum velocity to mean velocity for laminar Poiseuille fully developed flow and blunt flow are 2.0 and 1.0, respectively. In addition, significant velocity fluctuations were measured at the center of the pipe as shown in Fig. 3. The root mean square for velocity traces near the centerline was approximately 3 cm/s for both Reynolds numbers. This corresponds to fluctuations that are 7.5 and 6.25% of the mean centerline velocity for Reynolds numbers 2200 and 3000, respectively. Thus, the flow is not laminar at these Reynolds numbers and is in transition to turbulence.

**Figure 4:**
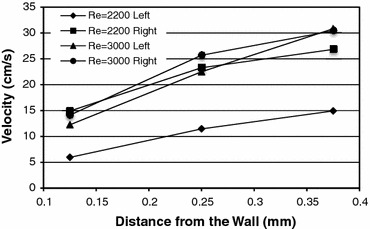
Near wall velocity distribution obtained from LDA used to calculate WSS

The near wall velocity measurements are shown in Fig. 4. Velocity gradients near the wall can be estimated using the two points nearest the wall. The velocity gradients for Reynolds number 2200 on the left and right walls are 442 and 670 s^−1^, respectively. The velocity gradients for Reynolds number 3000 on the left and right walls are 820 and 926 s^−1^, respectively. Under an assumption that the eddy viscosity is negligible, WSS was estimated as the product of the velocity gradient and viscosity. For Reynolds number 2200, the WSS estimate is 2.9 and 4.4 dynes/cm^2^ for left and right walls, respectively. For Reynolds number 3000, it is 5.3 and 6.0 dynes/cm^2^ for left and right walls, respectively. As the flow is transitional, we expect the eddy viscosity to contribute to the time averaged WSS. These values are greater than the theoretical values for laminar flow (2 and 2.8 dynes/cm^2^) and near the values obtained from the Moody diagram for turbulent flow (3.7 and 6.1 dynes/cm^2^). The WSS values and the velocity profile are more symmetric at Reynolds number 3000 than for Reynolds number 2200. This is due to the enhanced momentum transfer at higher Reynolds numbers resulting in the flow requiring less pipe diameters to be fully developed or symmetric. Thus, WSS is estimated for Reynolds number 2200 and 3000 in this cylindrical transitional flow system to be between 3–4 and 5–6 dynes/cm^2^, respectively.

### Effect of Shear Stress Under Transitional Fluid Flow on Cell Morphology

**Figure 5:**
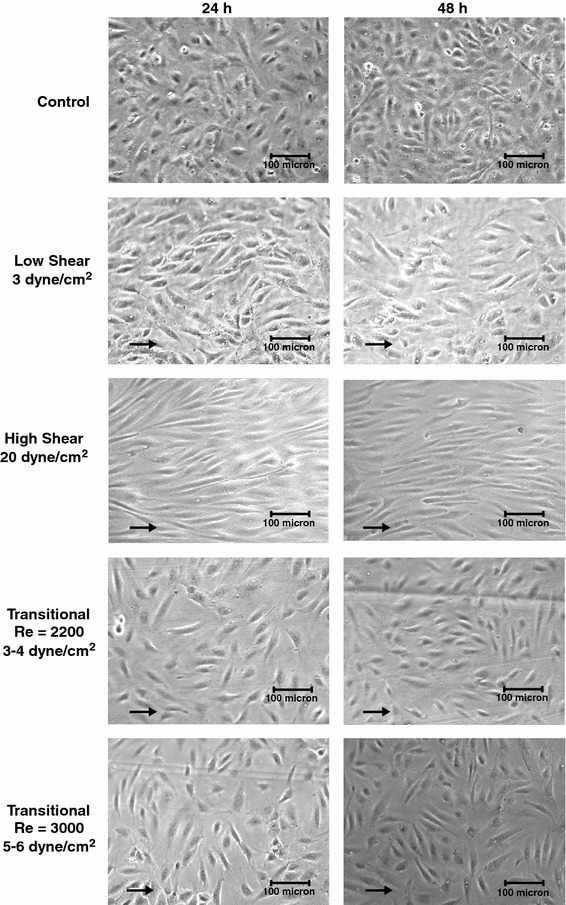
Brightfield images of ECs exposed to no shear stress (Con), 3 and 20 dyne/cm^2^ laminar shear stress, and shear stress due to transitional flow with Reynolds numbers of 2200 (3–4 dyne/cm^2^) and 3000 (5–6 dyne/cm^2^) at 24 and 48 h. Arrows point in the direction of fluid flow. Under laminar shear stress the cells have elongated relative to control cells and have aligned with the direction of flow in a magnitude dependent manner. Under transitional flow the cells are randomly organized as they are in the absence of fluid flow but they have elongated

**Fig. 6:**
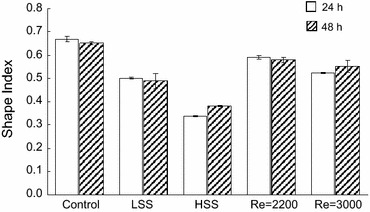
Mean shape index values of ECs exposed to no shear stress (Control), 3 and 20 dyne/cm^2^ laminar shear stress (LSS and HSS, respectively), and shear stress due to transitional flow with Reynolds numbers of 2200 (3–4 dyne/cm^2^) and 3000 (5–6 dyne/cm^2^) for 24 and 48 h

To determine the effect of transitional shear stress on morphology, confluent monolayers of ECs were exposed to shear stresses under transitional flows with Reynolds numbers of 2200 and 3000 for 24 and 48 h (Fig. 5). For comparison purposes, cells were also exposed to low and high laminar shear stresses of 3 and 20 dyne/cm^2^, respectively. Under low laminar and transitional flow, the cells were exposed to approximately the same shear stress magnitude. In agreement with previous reports, cells exposed to laminar shear stress elongated significantly relative to cells cultured in the absence of shear stress (controls) (Fig. 6). The elongation was magnitude dependent as cells exposed to high shear stress were significantly more elongated than those exposed to low shear stress (*p* < 0.05). At 24 and 48 h the shape index of cells exposed to 20 dyne/cm^2^ was 0.34 ± 0.01 and 0.38 ± 0.01, respectively, whereas it was 0.50 ± 0.01 and 0.49 ± 0.03 for 3 dyne/cm^2^. For both transitional shear stresses, the cells were significantly more elongated than control cells at 24 and 48 h (*p* < 0.05, Fig. 6). At 24 h the cells exposed to 3000 Reynolds number flow had elongated more than those at 2200 (*p* < 0.05), but at 48 h there was no significant difference. Relative to cells exposed to laminar shear stress, cells exposed to both transitional shear stresses were rounder than those exposed to high shear stress at 24 and 48 h (*p* < 0.05). At Reynolds number 2200, the cells were rounder than those exposed to low laminar shear stress (*p* < 0.05), whereas at 3000 there was no significant difference compared to low laminar shear stress at 24 and 48 h.

**Fig. 7:**
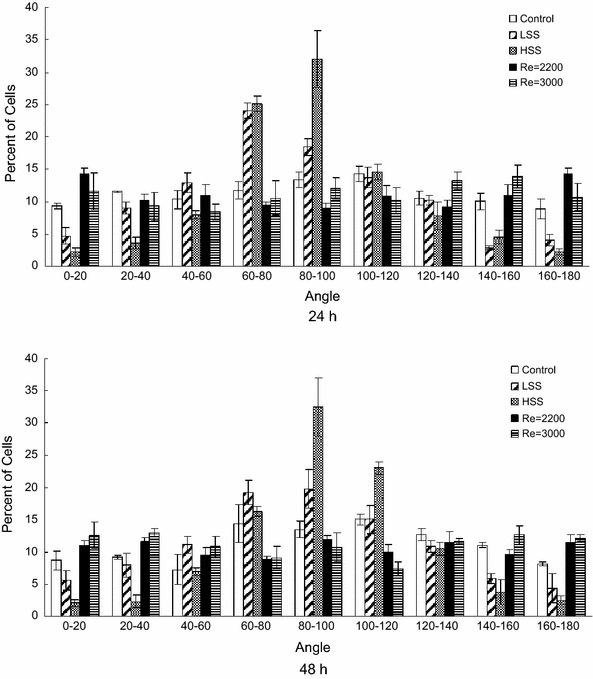
Angle frequency distributions of ECs exposed to no shear stress (Control), 3 and 20 dyne/cm^2^ laminar shear stress (LSS and HSS, respectively), and shear stress due to transitional flow with Reynolds numbers of 2200 (3–4 dyne/cm^2^) and 3000 (5–6 dyne/cm^2^) for 24 and 48 h. Graphed are the mean values of three independent experiments. Cells at 90° were aligned parallel to the direction of flow and cells at 0 and 180° were aligned perpendicular to the direction of flow

The degree with which the cells aligned with the direction of flow was determined as a second measurement of transitional flow’s effect on cell morphology. Histograms of the angles between the major axis of the cells and a line perpendicular to flow, in 20° increments are shown in Fig. 7. Using these coordinates, a cell with an angle of 90° will be aligned parallel to the direction of flow. In agreement with previously reports, the cells exposed to laminar shear stress showed alignment with the direction of flow, as the cells were centered around 90°. In sharp contrast, the cells exposed to transitional shear stress showed no alignment relative to the direction of flow, as their angles were equally distributed between 0 and 180° (Fig. 7).

**Fig. 8:**
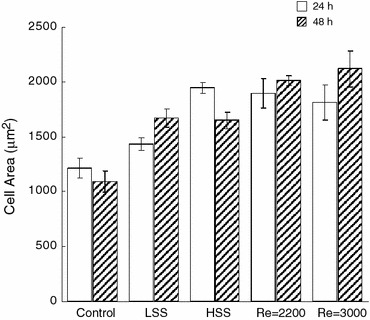
Mean cell area of ECs exposed to no shear stress (Control), 3 and 20 dyne/cm^2^ laminar shear stress (LSS and HSS, respectively), and shear stress due to transitional flow with Reynolds numbers of 2200 (3–4 dyne/cm^2^) and 3000 (5–6 dyne/cm^2^) for 24 and 48 h

The third parameter of cell morphology that was measured was cell area. Cells exposed to transitional shear stress were significantly more spread out than control cells after 24 and 48 h of exposure (*p* < 0.05, Fig. 8) with the cells spreading equally under both flow patterns. In comparison to laminar shear stress, cells cultured under flow with a Reynolds number of 3000 were more spread than those exposed to low and high laminar shear stress at 48 h (*p* < 0.05).

## Discussion

The cylindrical flow system presented here allows transitional shear stress studies at the cellular level. This system allows Reynolds number flow from 1000 to 3000, which is the same as that reported for AVGs *in vivo.* 13,38 Previously, systems were designed for the exposure of cells to laminar disturbed flow.^6^,^8^,^46^,^47^. The present design provides a transitional flow environment similar to that found in AVGs *in vivo*. Detailed experimental and computational studies in AVGs have been conducted using subject specific *in vitro* models to describe the AVG hemodynamic environment using an animal model.^22^,^24^,^25^,^30^ These studies show the flow environment to be transitional at Reynolds numbers as low as 800 but also laminar up to at least 1400 depending on the flow conditions. For AVGs under laminar flow,^25^ the velocity distributions inside of the venous anastomosis and downstream of the anastomosis were very different from those for flow through a straight pipe. Opposite of the graft entrance, a strong velocity jet existed providing axial WSS six times higher than Poiseuille flow through a tube of similar diameter and flow rate. In contrast, the WSS on the graft side wall was almost zero. As the jet convected downstream it swirled into an irregular pattern and was highly affected by the slight irregularities in the vein wall shape providing almost random regions of high and low WSS. These results indicate that exposure of cells to laminar flow within a cylindrical tube would not provide a similar flow environment to that found in AVGs *in vivo*. However, for AVGs under turbulent flow,^25^ the distributions of velocity and WSS (average and fluctuating) were more uniform downstream of the anastomosis due to the enhanced momentum transfer. These results indicate that exposure of cells to transitional flow within a cylindrical tube would provide a flow environment similar to that found in AVGs just downstream of the anastomosis. Under the current flow conditions, the computed WSS values obtained from LDA measurements (3–6 dyne/cm^2^) were lower than that present in an AVG *in vivo* (90 dyne/cm^2^). This difference in shear stress magnitude is caused by the more than five fold lower viscosity of media compared to that of blood. In order to maintain the same Reynolds number, the flow rate in the system was lowered by the same ratio. The viscosity of the media could be increased in future experiments by adding dextran to the media.^18^,^31^ However, these transitional flow experiments would not be feasible with a gravity driven flow system due to the increased pressure drop caused by a higher viscosity. In our experiments we specifically wanted to expose cells to laminar shear stress with the same magnitude as that of the transitional flows. A goal of this paper was to test the hypothesis that ECs would respond differentially to transitional and laminar shear stress due to the type of flow present. By having the transitional shear stress between 3 and 6 dyne/cm^2^, we were able to compare these results with those of our laminar flow system at 3 and 20 dyne/cm^2^.

The Reynolds numbers for the two different flow rates are easily and accurately quantified to be 2200 and 3000. However, quantification of the WSS the cells are exposed to is more difficult. Transitional flows are inherently less predictable than either laminar or fully turbulent flow regimes as described in detail by Natrajan and Christensen.^35^ While their study detailed transitional flow within a pipe with diameter of 536 *μ*m, their results using particle image velocimetry demonstrate the complex flow patterns of oscillating vorticies that can be present in transitional flows. The effect of these flow patterns on the instantaneous WSS can be significant for these non-laminar flows. While the WSS cannot be perfectly described for this flow system, WSS estimates can be obtained. A limitation of this system is that the entrance length was not sufficient to produce fully developed laminar flow although it was sufficient for turbulent flow at these Reynolds numbers. There is also sudden change in the diameter of the system from 6 to 5.74 mm due to the placement of the polycarbonate sheet (thickness 0.13 mm). In addition, we don’t know the precise surface roughness values for the ECs although we have an estimate from measurements by Liu *et al*.^28^ Finally, the velocity profile at Reynolds number 2200 is slightly skewed which can alter the WSS magnitude circumferentially. Thus, a precise value of the time averaged WSS the cells are exposed to is difficult to obtain. However, based on WSS estimates obtainable from the experimental measurement of velocity gradient, laminar flow theory, and turbulent flow data for these flows, we can provide a range of WSS values that the cells were likely exposed to. The estimates as described previously are between 3 and 4 dynes/cm^2^ for Re = 2200 and between 5 and 6 dynes/cm^2^ for Re = 3000. There may be cells exposed to WSS values outside of this range on the order of ±1 dynes/cm^2^, however, we expect that to be a small percentage of the cells, likely near the edge of the polycarbonate sheet. While WSS values for the laminar and transitional flows did not match exactly, it is unknown if ECs can sense such a small WSS difference.

Prior to exposure to shear stress in the transitional system, the cells are cultured on a thin piece of polycarbonate film. This is advantageous over directly culturing the cells in the tube for several reasons. First, it is easier to homogenously seed and culture the cells prior to flow. It is difficult to homogenously seed cells within cylinders as the cells tend to attach unevenly and clump together. This would also require the chamber to be rotated, which would expose the cells to shear stress. Second, since the film can be removed prior to cell lysing, greater percentages of RNA and protein are recovered for biological assays. Third, it is easier to image the cells with a microscope before and after exposure, as the film can be laid flat for imaging.

The cylindrical flow system was used to study the effects of shear stress under transitional flow on cell morphology. We also exposed ECs to laminar shear stress for comparison. The culturing and seeding conditions for the cells exposed to laminar and transitional shear stress were the same. In both cases, cells were cultured on a polycarbonate substrate coated with fibronectin at a density of 5.0 × 10^4^ cells/cm^2^. This was necessary since it has been shown that the effect of shear stress on cells is dependent upon the cells’ substrate and confluency.^27^,^37^ As expected and shown by many others, the cells exposed to laminar shear stress elongated and aligned with the direction of flow in a magnitude dependent manner (Figs. 6, 7). What was unexpected was the elongation of the ECs exposed to transitional shear stress. The present results show that cells exposed to transitional flow were more elongated than control cells (Fig. 7). This is in contrast to the findings of Davies *et al*.^7^ experiments, where cells in turbulent cone-and-plate flow remained cuboidal in shape. At Reynolds number 2200, the cells did not elongate to the extent of those exposed to low and high laminar shear stress. However, at a Reynolds number of 3000, the shape indexes were not significantly different from those of low laminar shear stress at 24 or 48 h. Transitional shear stress caused the cells to elongate at Reynolds number 3000 similar to laminar flow. However, transitional shear stress did not cause cell alignment in contrast to laminar flow. This could be due to small fluctuations in the WSS vector direction away from axial, which occur in transitional flows. These fluctuations may have been inhibitory for cell alignment but not elongation. In conclusion, this transitional flow chamber provides a novel method for studying the effect of transitional shear stress on cells. Transitional shear stress caused elongation but not the alignment observed under laminar shear stress. Additional experiments are necessary to determine the mechanical signaling pathways that are activated under transitional flow conditions.
